# Wish to die trying to live: unwise or incapacitous? The case of University Hospitals Birmingham NHS Foundation Trust versus ‘ST’

**DOI:** 10.1136/jme-2024-110365

**Published:** 2024-11-14

**Authors:** Johnna Wellesley, Dominic Wilkinson, Bryanna Moore

**Affiliations:** 1 Bioethics and Health Humanities, University of Texas Medical Branch Office, Galveston, Texas, USA; 2 Uehiro Oxford Institute, University of Oxford, Oxford, UK; 3 Newborn Care, John Radcliffe Hospital, Oxford, UK; 4 Centre for Biomedical Ethics, National University of Singapore Yong Loo Lin School of Medicine, Singapore; 5 Murdoch Children’s Research Institute, Melbourne, Victoria, Australia; 6 Department of Paediatrics, School of Clinical Sciences, Faculty of Medicine Nursing and Health Sciences, Monash University, Melbourne, Victoria, Australia; 7 Department of Health Humanities and Bioethics, University of Rochester, Rochester, New York, USA

**Keywords:** Decision Making, Ethics- Medical, Terminally Ill

## Abstract

The recent legal dispute about medical treatment for a 19-year-old patient, Sudiksha Thirumalesh, (known initially by the Court of Protection as ‘ST’) in A NHS Trust versus ST & Ors (2023) raised several challenging ethical issues. While Sudiksha’s case bears similarities to other high-profile cases in England and Wales, there are key differences. Crucially, Sudiksha herself was part of the disagreement. She was alert, communicative and sought to advocate for herself. Furthermore, this case was framed in the courts as pivoting not on considerations of best interests but on a determination of decisional capacity. Sudiksha was deemed to lack capacity because she did not believe her doctors’ view of her prognosis.

While the legal questions in the case were central to a recent Court of Appeal decision (which overturned the original finding), in this commentary, we focus on the ethical questions therein. We start by describing Sudiksha’s court case and the initial judgment. We then offer an ethical analysis of the relationship between false beliefs, values and the ‘capacity’ to make decisions, arguing for a need for particular care when judging patients to lack capacity based purely on ‘false and fixed beliefs’. After briefly noting the legal basis for the appeal finding, we offer ethical implications for future cases. Although it appears that Sudiksha had decision-making capacity, this did not settle the ethical question of whether health professionals were obliged to continue treatment that they believed to have no prospect of success.

## Introduction

### The case

 In the summer of 2022, Sudiksha Thirumalesh, known initially by the Court of Protection as ‘ST’, was admitted to hospital with respiratory arrest resulting from a rare mitochondrial condition as well as a positive COVID-19 infection. She spent the next year being cared for in the intensive care unit. She was dependent on mechanical ventilation, artificial nutrition and regular haemodialysis and experienced health issues including ‘impaired sight and hearing loss, chronic muscle weakness, bone disease and chronic damage to her kidneys and lungs’^1^ (para 1). The NHS Trust overseeing her care argued that Sudiksha’s disease was progressive and would lead to her death. They proposed a transition to palliative care that included discontinuing haemodialysis but maintaining mechanical ventilation and artificial nutrition. This plan was opposed by Sudiksha and her family who expressed a desire to explore her eligibility for experimental treatments at another facility in the USA or Canada. Sudiksha expressed hope that she might return to school to complete her A-levels. While she accepted that ‘her chances of some recovery were no more than 50%’ (para 5), she also insisted, ‘This is my wish. I want to die trying to live. We have to try everything’ (para 4).

The key issue brought before the Court of Protection on 12 August 2023 was Sudiksha’s capacity to make decisions regarding future treatments. It was Sudiksha’s view that her respiratory condition was a result of long COVID and not the progression of mitochondrial disease. She believed she would recover with experimental nucleoside treatment overseas. The Trust submitted that Sudiksha was actively dying. They argued that she lacked decision-making capacity: Sudiksha did not believe the facts pertaining to her disease including her poor prognosis and was thus unable to make a decision about treatment.

### Views of the experts

In England and Wales, there is a two-pronged test for (in)capacity under the Mental Capacity Act 2005 (‘MCA 2005’).^2^ Stage 1 asks whether the person is unable to make a particular decision (the functional test) and stage 2 asks whether they are unable to make a decision because of an impairment of or disturbance in the functioning of a person’s mind or brain. This could be due to long-term conditions such as mental illness, dementia, learning disability or more temporary states such as confusion, unconsciousness or the effects of drugs or alcohol (the ‘impairment’ or diagnostic test).

Dr A, the critical care physician in charge of Sudiksha’s care, expressed concern about Sudiksha’s ‘deeply seated misunderstanding’ (para 29) of the severity of her condition and the inevitable deterioration of her respiratory function. Dr A felt that this rendered her unable to weigh her options, specifically palliative care. In terms of her prognosis, though an exact timeline was uncertain, Dr A estimated a 25% chance of survival to the autumn. Dr E, a paediatric specialist in mitochondrial disorders, agreed with Dr A’s estimated timeline and felt that Sudiksha’s disease burden was too great to benefit from nucleoside intervention and she would continue to experience major organ decline. In all other respects, Dr A felt Sudiksha could engage in capacitous decision-making about her day-to-day as the elements of those decisions were not impacted by her ‘delusion from a false reality’ (para 31).

Drs C and D, two consultant psychiatrists, found no psychological disturbance or misfunctioning of the brain when they assessed Sudiksha. Furthermore, Dr C stated that Sudiksha’s beliefs which she shared with her family were not irrational but grounded in an understandable hope to stay alive as long as possible. Dr C concluded that she had the capacity to make decisions regarding her treatment; however, she may not meet all elements of the functional test because Sudiksha did not demonstrate an understanding of certain aspects of her medical circumstances. She could not, therefore, weigh the consequences of future decisions. Dr C described Sudiksha’s view of the probability of her own recovery as a ‘fixed and false belief’ (para 50(i)).

Dr D’s submission was that Sudiksha was neither delusional nor suffering from false belief but that she held a ‘different opinion’ (para 42) about the imminence of her death. In his view, she did not fail the functional test of the MCA as her brain and mind functioning were intact.

### The judgment

In August 2023, Justice Roberts concluded that Sudiksha’s refusal to believe what the clinical team told her about her prognosis and the impossibility of travelling overseas meant that she was unable to weigh treatment decisions properly and thereby lacked decision-making capacity. Although she did not have a formally diagnosed psychiatric or psychological illness, her long period of critical illness and young age were thought to have affected her ability to make decisions and to deal with the considerable stress she was experiencing. Importantly, no decision was made about treatment—that was to be adjudicated at a subsequent hearing. However, Sudiksha died from cardiac arrest on 12 September 2023 before any subsequent hearing could take place.

In May 2024, the Court of Appeal heard an appeal against the judgment of incapacity.^3^ Although the results of the appeal could not benefit Sudiksha, the Court agreed to hear it because of the wider significance of the issues raised.

## Ethical issues with the judgment

This case raises several ethical issues. We focus here primarily on the normative questions that underlie the controversial role of ‘belief’ in the assessment of a patient’s capacity to make medical decisions, particularly in situations where a patient does not believe information conveyed to them by their doctor. The statutory requirements of the MCA framed the ethical challenges in this case but while we draw on the late Justice Roberts’ initial judgment, our focus is primarily ethical rather than legal. After summarising our own ethical analysis of the case, we will briefly summarise the legal argument in the Court of Appeal.

### Belief and disbelief

The different elements of the functional test for capacity include the ability to understand, weigh and retain the information relevant to the decision as well as communicate a response. In Sudiksha’s case, the focus was on belief as a necessary requirement for understanding. Roberts J noted in the judgment that ‘If one does not ‘believe’ a particular piece of information then one does not, in truth, ‘comprehend’ or ‘understand’ it nor can it be said that one is able to ‘use’ or ‘weigh’ it. In other words, the specific requirement of belief is subsumed in the more general requirements of understanding and ability to use and weigh information’ (para 81). However, there are at least three reasons for being sceptical about this basis for judging a patient to lack capacity.

#### Beliefs are in part evaluative

Understanding and weighing necessarily include cognitive (technical) elements. It makes sense to include those cognitive elements within an assessment of capacity. For example, someone who was unable to understand what it means to die would not be able to make a decision about accepting or refusing a life-prolonging treatment. However, understanding and weighing information also includes evaluative elements such as the value placed on certain outcomes. Crucially, values can have epistemic components that influence what you choose to believe and even possibly what or who you are able to believe. However, a patient’s values are not the sort of thing that ethically justifies overruling their choices. Indeed, respecting autonomy fundamentally requires allowing patients to develop beliefs and make choices in line with their values.

As an example of how values can affect beliefs, hope can and often does play a significant, influential role in decision-making. Blumenthal-Barby and Ubel define hope as occurring ‘when a person has a desire for a certain outcome, believes the desired outcome is possible (probability is greater than zero), and engages in certain behaviours related to it such as praying for it, thinking or fantasising about it, or even planning for it.’^4^ A patient’s hopes may not be shared by health professionals. Unrealistic optimism, by contrast, occurs when ‘a person has a desire for a certain outcome and overestimates the probability of the desired outcome’. Denial occurs ‘when a person has a desire for a certain outcome and fails to think about or ‘face’ the high probability of the undesired outcome’.

However, neither hope, unrealistic optimism, denial nor even false belief is necessarily indicative of a lack of decision-making capacity. Someone’s false belief could arise from an impairment of the functioning of their brain and ability to reason but it could also arise as a function of their values. That is most clearly evident when it comes to religious beliefs. In such cases, a patient’s values (the things to which they attach importance) affect what they choose to believe. It can occur substantively—for example, a belief in life after death, the power of prayer or the likelihood of miraculous intervention.

#### Beliefs are (at least in part) chosen

It is important to distinguish between situations where someone is (1) capable of believing X but chooses not to do so and (2) where they are (literally) incapable of believing X. The latter would arguably ground a judgement of incapacity. A patient who has severe persecutory delusions or who has been brainwashed may be unable to believe the information given to them by an authority figure (eg, a doctor). But the former is clearly different: A chosen belief (or disbelief) should not be the ethical basis for excluding someone’s decision-making ability. For example, a vaccine sceptic may disbelieve the information offered by all health professionals. But that does not make them incapable of deciding to have (or, more likely, refuse) a vaccine. (There are important philosophical debates about doxastic voluntarism and the degree to which our beliefs are or can be under our control.^5 6^ We set those issues aside for this paper. For present purposes, we assume the (relatively) uncontroversial claim that at least some of our beliefs are at least partly under our voluntary control.)

It may be that Sudiksha was literally incapable of believing that she would die. Based on the available evidence, however, this does not seem like the case. Sudiksha appeared to demonstrate an understanding of the information provided to her and recognise its significance. Unlike other patients in intensive care who are sedated and unable to communicate, Sudiksha engaged with the clinical team. She was able to articulate her wish to explore treatment options that would allow her to be weaned from ventilator dependency. She acknowledged the decline in her condition as well as the ‘untried and untested’ (para 55) treatments potentially available abroad. Her stated desire to ‘die trying to live’ appeared to indicate a recognition that at some point in the future, she may succumb to her condition and die (para 84).

Sudiksha’s treating clinician (Dr A) gave evidence that Sudiksha had ‘closed her mind to the alternative of ‘greater comfort’…which palliative care is likely to provide’ (para 29). Roberts J observed that she ‘refuses to contemplate’ that the prognosis given to her by her doctors was true (para 86) and referred to her ‘fundamental distrust in, and refusal to accept’ the information provided (para 94). The language used by the judge and consultant is telling here. By direct implication, someone who ‘closes their mind’ or ‘refuses to believe’ is engaging in a voluntary doxastic act. In Sudiksha’s case, those actions seem to have been motivated by her ‘overwhelming desire to survive’ (para 84). But this highlights another way in which belief is more than a cognitive process. Where beliefs are shaped by patient desires that strengthens the case that these are partly an expression of (and not a threat to) their autonomy. Accepting Sudiksha’s beliefs, then, may have been perfectly consistent with the ethical principle of respect for autonomy.

#### Shared beliefs imply shared capacity (or incapacity)

Where a patient’s beliefs are idiosyncratic, that might indicate that there is something amiss in their ability to understand relevant information. However, where beliefs are shared with others, that provides at least some evidence in support of the belief being value-based and chosen rather than arising from a disorder of the mind.

In Sudiksha’s case, both the judge and the consultant psychiatrist referred to the overlap between Sudiksha’s beliefs and those of her family including their ‘refusal to accept’ and ‘believe’ the information from the doctors. In the opinion of Dr C, Sudiksha’s ‘failure to understand the nature of her illness and her consequent ability to weigh up the facts in a decision-making process is not because of an impairment of mind but rather the result of the shared beliefs that she holds with her family’ (para 53). There was no suggestion that Sudiksha was inappropriately influenced by her family’s beliefs but rather the implication was that the family as a whole simply shared a strong belief in the possibility of recovery.

Of course, the fact that other people hold a certain belief does not mean that they are right (they may all be mistaken) or even that they are all capable of reasoning (they may be suffering from a group delusion). But this points to a fundamental challenge in Sudiksha’s case. If Sudiksha was judged to lack decision-making capacity on the basis of a fixed false belief about her prognosis then it would follow that anyone with similar beliefs who made the same decision as Sudiksha in her situation would also lack capacity. However, a reverse inference is more plausible. It appears highly unlikely that other individuals who shared Sudiksha’s beliefs would be judged to ‘lack capacity’ to make those decisions about medical treatment. To outside observers, Sudiksha and her family’s hope against hope may seem rational and understandable. Apart from anything else, there would be no basis for thinking that the others who held such views (ie, Sudiksha’s family) suffered from an impairment of the functioning of their mind or brain. If those others would be regarded as having the capacity to make this decision then it seems only reasonable to think that Sudiksha also had the capacity.

## Legal appeal

On 31 July 2024, the Court of Appeal, in a landmark ruling restored the presumption of capacity in Sudiksha’s case. Lady Justice King, in para 138, ruled that:

Once one displaces an absolute requirement for ‘belief’, then, where a 19-year-old young woman, fully conscious and suffering no identifiable mental illness or loss of brain function and with the full support of her close-knit family, refuses to accept that her death is imminent but says loud and clear to two psychiatrists that she wants to ‘[d]ie trying to live’, it will take a great deal to displace the principle of autonomy and the presumption of capacity, no matter how unwise her decision to eschew palliative care may have seemed to a more mature mind.

Both sides of the Appeal agreed that the false belief may impact someone’s ability to understand and use or weigh information. However, the appeal judges determined that Sudiksha’s beliefs, while entailing a mistaken understanding of the clinical reality, were not grounds for incapacity. There were complex legal arguments, but in essence, the Court of Appeal judges concluded on the basis of relevant case law and the MCA that belief is not a separate and determinative component of capacitous decision-making but rather is subsumed within a broader evaluation of Sudiksha’s understanding. King LJ noted Butler-Sloss LJ in *Re MB*:^7^ ‘An absence of belief may but not inevitably will, on the facts of a particular case, lead to a clinician or a court to conclude … that the person in question does not have the ability to make the decision in question’ (para 123). In other words, patients can, in some cases, understand the relevant information but not believe it and still retain the capacity to decide. The appeal judges were also critical that in the original judgment, Roberts J had rejected the evidence of two court-appointed psychiatric experts and preferred that of the attending clinicians without providing reasons for that preference. Importantly, we think that Lady Justice King’s judgment better aligns the court’s position with prevailing ethical norms and guidance.

## Ethical implications for future cases

While capacity assessments involve some critical inquiry into a person’s values and beliefs, they are primarily procedural in nature; that is, they concern the process of someone’s ability to understand, weigh and balance information and reason—not the content of someone’s values or beliefs. The original court’s judgment in Sudiksha’s case seemed to entail both a judgement that she could not manipulate information in the necessary way and that her beliefs prevented her from being able to make a decision. However, basing capacity assessments on judgements about the content or substance of someone’s beliefs/values is ethically fraught and may undermine principles of personal autonomy and self-determination, especially in pluralistic societies. Patients sometimes have culturally or religiously informed beliefs that motivate their acceptance or refusal of certain treatments. The consequences of requiring patients to hold beliefs that concord with those of one’s treating clinicians in order to have decision-making capacity would be far-reaching. To impose the beliefs of a certain individual or group on patients undermines the professional standards of medical practice and care which require partnership with patients and families taking into consideration their values and preferences.

Although we have argued that the original decision in Sudiksha’s case was ethically flawed and the Court of Appeal found the decision to be legally flawed that does not necessarily resolve the question of what clinicians should do in cases where patients refuse to accept they are approaching the end of their lives and wish to continue with aggressive or experimental treatments.

A key ethical and legal principle is that a patient with capacity may refuse any (or indeed all) medical treatment. As a consequence, given that Sudiksha appeared to have capacity, she should have been free to decline the recommended palliative care. However, patients’ right to refuse treatment does not translate into a right to insist that treatments are provided or must continue. While there can be challenges associated with identifying when treatment is physiologically futile or non-beneficial, health professionals are ethically entitled to decline to provide treatment that they believe would be contrary to a patient’s best interests or would be an inappropriate use of limited resources.^8 9^

Since there is no ethical difference between withdrawing and withholding treatment, in principle, it may have been appropriate for Sudiksha’s clinicians to indicate that they were not willing to continue treatment such as haemodialysis. However, in practice, it can be extremely challenging to unilaterally withdraw treatment on the basis of a best interests determination or limited resources and there is no clear legal precedent for doing so for patients with capacity outside of crisis standards of care.^10^ Of note, there were potentially other legal pathways open to the treating clinicians and courts in Sudiksha’s case. They might have drawn on the so-called ‘inherent jurisdiction’ of the High Court. This provides a legal mechanism to protect adults who have capacity (and are therefore outside the jurisdiction of the Court of Protection and the MCA) but who are vulnerable in one or more ways. Justice Munby in a much-cited passage previously clarified that “the inherent jurisdiction can be exercised in relation to a vulnerable adult who, even if not incapacitated by mental disorder or mental illness, is or is reasonably believed to be, either (1) under constraint or (2) subject to coercion or undue influence or (3) for some other reason deprived of the capacity to make the relevant decision or disabled from making a free choice or incapacitated or disabled from giving or expressing a real and genuine consent”.^11^ It is unclear what conclusion the High Court would have reached if it had been asked. Since the underlying values and principles significantly overlap, the same decision might have been reached. However, in our view, pursuing such other legal pathways rather than focusing on Sudiksha’s capacity would have been more appropriate.

Sudiksha’s case also raises questions regarding the role of expert testimony where there is doubt or disagreement about a patient’s capacity. There are interesting procedural and practical issues at stake for patients in these constrained and vulnerable situations. Adult patients are normally presumed to have decision-making capacity until they demonstrate otherwise but this is not the default position for adolescents. In Sudiksha’s case, once the dispute arose, it seems to have been her responsibility to demonstrate she had capacity in a series of assessments with different experts—there was no presumption of capacity.

## Conclusion

In this paper, we have argued that there is a need for more careful analysis of the relationship between beliefs, values and the ‘capacity’ to make decisions, especially when judging a patient to lack capacity based purely on ‘false and fixed beliefs’. Although we have analysed Sudiksha’s case on the basis of information available in the published legal judgments, we do not have access to all of the medical or personal details. Ultimately, we can only speculate about her state of mind, understanding and decision-making. It may be that Sudiksha’s values were influenced by both her family and her own religious orientation (though this was never central to the argument) and she was thus either unable or unwilling to believe certain negative prognostic information. Or it may be that her doctors had been wrong about her prognosis on previous occasions and thus created grounds for reasonable doubt or even mistrust in the word of experts. Or it may be that Sudiksha’s request to ‘die trying to live’ was just that—a judgement about what, for her, was an acceptable way to die or a ‘good’ death (akin to Dylan Thomas’ famous injunction to his dying father).^12^ For Sudiksha, there appeared to be a significant overlap between hope, reasonable belief and rationality that contributed to her own process of understanding and weighing the information given to her by her doctors. We have argued that decisional capacity ought to turn on the technical (cognitive) elements and not the evaluative elements. Given the subjective and variable nature of someone’s beliefs and values, attempts to measure these evaluative elements in capacity determinations are inherently risky.

Finally, we have noted that a determination of capacity does not exhaust ethical considerations or legal options in disputes about medical treatment for adults. There can be separate, strong ethical reasons that can justify limiting treatment (for example, on the basis of limited resources). There is a need to be clear and honest about those reasons if they are to be the basis for decisions.
